# Comparative investigation of the structural characteristics of tobacco stalk lignin during the DES and alkaline deconstruction toward sustainable materials

**DOI:** 10.3389/fbioe.2022.994760

**Published:** 2022-08-25

**Authors:** Na Wang, Bo Wang, Hui Si, Suxia Hu, Lin Chen, Yu Liao, Lei Wang, Yifan Zhang, Jungang Jiang

**Affiliations:** ^1^ China Tobacco Hubei Industrial Co., Ltd., Wuhan, China; ^2^ Hubei Provincial Key Laboratory of Green Materials for Light Industry, Hubei University of Technology, Wuhan, China

**Keywords:** macromolecular structure, tobacco stalk, lignin, deep eutectic solvent, UV absorption

## Abstract

Lignin polymer as a natural aromatic macromolecule presents significant prospects in producing functional and sustainable materials, and achieving a comprehensive characterization will facilitate their target valorization. In the present study, deep eutectic solvent (DES) and alkaline delignification were adopted to deconstruct tobacco stalk before and after hydrothermal pretreatment, obtaining diverse lignin fractions with fascinating characteristics. DES lignin exhibited a higher yield and homogenous molecular structure than MWL. A severe cleavage of the inter-unit linkages in lignin was also observed. This result mostly originated from the efficient delignification of the DES deconstruction system adopted. Moreover, all the recovered lignin fractions exhibited good micro-nanoparticle size that can enhance the valorization of lignin in nanomaterial production, in which the hydrothermal-assisted DES deconstruction promoted the formation of the smaller lignin nanoparticle size. Next, all the recovered lignin presented an excellent UV absorption and structure-related absorption performance or thermal properties. Overall, this work provides an important foundation for further exploiting DES/alkaline delignification lignin that can be applied as an ideal feedstock for producing sustainable functional or micro/nanomaterials.

## Introduction

Lignocelluloses, mainly consisting of cellulose, hemicelluloses, and lignin, are considered fantastic alternative feedstock for the replacement of petrochemical resources, owing to their abundance, eco-friendliness, and renewability, which can be converted into diverse fuels, materials, and fine chemicals (Zhang et al., 2017; Liu et al., 2019). Lignocellulosic biomass is also an excellent resource for the cooperative and sustainable development of the global green economy. Tobacco stalk is a kind of typical waste biomass among various lignocellulosic feedstocks, which is generated from the agricultural industry (Wang et al., 2022). Tobacco stalk can be transformed into promising green energy and platform chemicals. However, this agricultural waste is always of low utilization, most of which is burned to generate heat, causing environmental pollution and wasting sustainable resources. Actually, lignin is an intricate heteropolymer that is cross-linked by various bonding motifs, for example, β-*O*-4, β-β, β-5, and β-1 (Ralph et al., 2004; Wang et al., 2017), and is a valuable resource that can be tailored into sustainable, functional materials (Laurichesse and Averous, 2014; Wang et al., 2020). However, the high recalcitrant and complex structures of tobacco stalk impede the utilization of components and conversion (Zhao et al., 2012). Thus, achieving effective deconstruction of lignocellulose is the principal step for lignin valorization.

According to the twelve principles of green chemistry, a biorefinery strategy should realize the high value-added utilization of biomass resources and adhere to the waste-free standpoint (Clarke et al., 2018). In recent years, some pretreatment methods such as physical, chemical, and biological pretreatment have been adopted to deconstruct different lignocellulosic materials (Clarke et al., 2018; Wang B. et al., 2018). Deep eutectic solvents (DESs) have attracted more and more attention as the potential green solvents for biorefinery with advantages such as easy synthesis, low cost, biodegradability, and low toxicity (Bjeli´c et al., 2020). DESs are generally prepared by hydrogen bond donors (HBDs) and hydrogen bond acceptors (HBAs), forming a stable and homogeneous solvent with desirable low cost and recyclability (Bjeli´c et al., 2020). At present, DES has been widely applied in the deconstruction of biomass, which can enhance the enzymatic hydrolysis rate of cellulose for sugar conversion and lignin dissociation (Hladnik et al., 2021; Wang et al., 2022). Among them, acidic and basic DESs exert efficient delignification toward various lignocelluloses, meanwhile neutral DES also showed great potential in lignin fractionation owing to its eco-friendliness and easy availability (Ma et al., 2021a; Wang et al., 2022). Alkaline DESs showed better structural retention for lignin macromolecules (Xu et al., 2021; Wang et al., 2022). However, the combination of glycerol (Gly) and choline chloride (ChCl) has an inferior ability to dissolve lignin (Alvarez-Vasco et al., 2016), whereas the addition of Lewis acids in the glycerol and choline chloride system could contribute to the satisfactory delignification of lignocellulose. This mainly originated from the proton-catalyzed linkage cleavage between lignin and matrix (Chen et al., 2020; Ma et al., 2021b). Actually, DES pretreatments can be adapted to isolate various lignin fractions that are promising materials for functional materials. Importantly, lignin extracted by the DES system can be utilized to fabricate different lignin-based materials, such as UV-blocking composite film, antioxidant polyethersulfone membranes, and lithium-ion batteries (Esmaeili et al., 2018; Guo et al., 2020; Vin-Nnajiofor. et al., 2022).

Recently, some studies have proved that polyalcohol-based DES systems, such as glycerol, ethylene glycol, and butanediol, exhibited ideal delignification efficiency for lignocellulose deconstruction (Chen et al., 2020; Cheng et al., 2022; Ma et al., 2022). It was reported that the isolation of lignin and the enhancement of enzymatic hydrolysis by various polyalcohol-based DES pretreatments were efficient by comprehensive investigation (Chen et al., 2018). These DESs can be used to enhance the production of high-yield fermentable sugars and tailored lignin nanoparticles and sustainably explore the strategy for the conversion of diverse biomass (Hladnik et al., 2021; Ma et al., 2022). Hydrothermal pretreatment (HTP), as an eco-friendly and economical technology, can not only improve hemicelluloses and obtain xylooligosaccharides (XOS) from biomass but also promote the deconstruction of lignocellulose (Wang et al., 2019a). The combination of HTP and DES is an excellent and green strategy for the valorization of lignocellulose, in which the structural characteristics of lignin during the deconstruction process are important for their value-added conversion into sustainable materials. Therefore, comprehensively investigating lignin structures is a precondition for lignin valorization. Alkaline delignification is a typical approach for deconstructing lignocellulose, and most of the industrial lignin that originate from this process is an ideal precursor for producing functional materials. Presently, no study has been performed to investigate the structural differences of lignin between DES pretreatment and alkaline delignification. Knowing their structural characteristics will guide the fabrication of lignin-based materials.

In the present study, DES deconstruction (ChCl–ethylene glycol–AlCl_3_·6H_2_O/ChCl–glycerol–AlCl_3_·6H_2_O) and alkaline delignification were applied to dissociate raw and hydrothermal tobacco stalk, acquiring different lignin fractions that were used to comparatively explore their structural features, such as lignin yield and lignin structures. Moreover, the structural changes of lignin during the DES pretreatment were comparatively elucidated by 2D-HSQC NMR and FTIR techniques compared to MWL from raw tobacco stalk. In addition, the micro-nano morphology and ultraviolet-visible light adsorption, as well as thermostability, were investigated. It is believed that the comprehensive characterization of lignin from DES deconstruction and alkaline delignification will boost the lignin valorization and further facilitate the exploitation of bio-based materials.

## Materials and methods

### Materials

Tobacco stalks were kindly provided by the Institute of Tobacco Science and Technology (Zhengzhou, China). Topically, tobacco stalks were cut into 3–5 cm length, then milled into 40–60 mesh powder, and oven-dried at 60°C for 48 h. Chemical compositions of the stalk are cellulose (40.5 wt%), hemicelluloses (21.5 wt%), lignin (22.3 wt%), and others (15.7 wt%), which are determined according to the National Renewable Energy Laboratory (NREL) method (Sluiter et al., 2008). Hydrothermal pretreatment (HTP) of tobacco stalk was performed in a stainless-steel reactor (Parr, United States) with a solid-to-liquid ratio of 1:8 (g/ml) at 170°C for 1 h (Wang et al., 2019b). Afterward, the treated tobacco stalks were separated by nylon gauze and oven-dried (60°C) to obtain HTP tobacco stalk. Choline chloride (ChCl), ethylene glycol (EG) or glycerol (Gly), aluminum chloride hexahydrate (AlCl_3_⋅6H_2_O), acetone (analytical grade), and sodium hydroxide (NaOH) were purchased from Sigma Chemical Co., Ltd.

### Preparation of DES

Two DESs were synthesized by mixing the pre-dried choline chloride (ChCl), ethylene glycol (EG) or glycerol (Gly), and aluminum chloride hexahydrate (AlCl_3_⋅6H_2_O) at a molar ratio of 1:2:0.1 under constant stirring at 80°C for 1 h (Ma et al., 2021a). The prepared transparent DES solvents were stored in a desiccator prior to use.

### DES pretreatment and alkaline delignification

**FIGURE 1:**
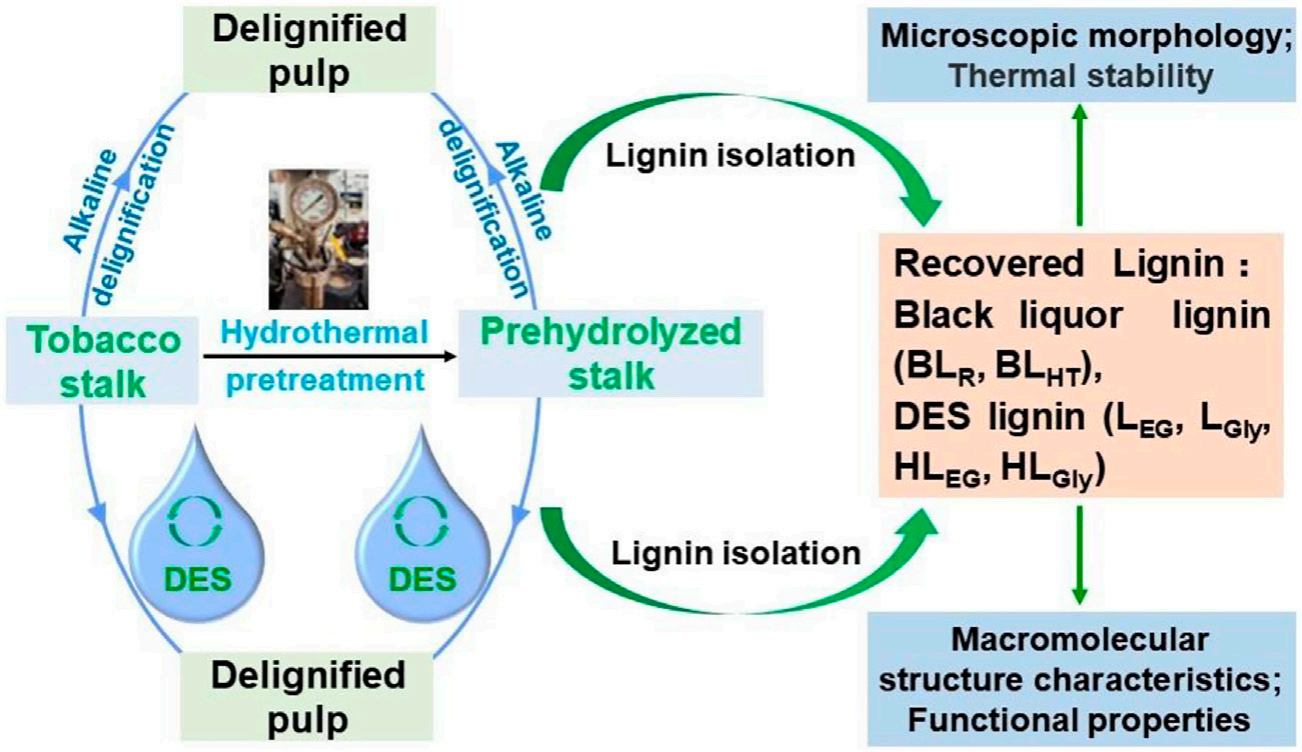
Scheme of tobacco stalk dissociation and lignin isolation.

The general scheme of deconstruction of tobacco stalk through DES pretreatment and alkaline delignification is depicted in Figure 1. In detail, the DES pretreatments of tobacco stalk were performed using a mixing ratio of 1:10 (w/w, tobacco stalk powder:DES) in a 50 mL round-bottom flask, which was heated in an oil bath raised (with a heating rate of 10°C/min) from room temperature to 120°C and held for 4 h. For HTP tobacco stalk, all the conditions were similarly adopted as mentioned earlier, except for the held time (2 h). All the conditions adopted in this work were based on previous studies (Ma et al., 2021b; Wang et al., 2022). After the DES pretreatment, acetone/water (50 ml, 1:1 v/v) solvent was poured into the resultant dark-brown liquid with stirring for 10 min to terminate the reaction. The mixture was vacuum-filtered and washed with the same acetone/water solvent three times to remove the residual DES. Then, all the filtrates were mixed and heated in a rotary evaporator at 50°C to remove acetone, and the recovered solids were centrifuged to obtain DES lignins, which were named L_EG_, L_Gly_, HL_EG_, and HL_Gly_, respectively.

Meanwhile, raw and HTP tobacco stalks were also subjected to alkaline delignification to obtain black liquor lignin (BL), aiming to comparatively investigate the structural characteristics between DES lignin and BL. Alkaline delignification of tobacco stalks was carried out under the following conditions: 20 wt% alkali charge (16 wt% for HTP tobacco stalks), 0.5 wt% anthraquinone (AQ) charge, solid-to-liquid ratio 1:5 (g/ml), and 140°C for 1 h (21). After cooking, black liquor and solid residue were separated by filtrating with a nylon bag, and the black liquor was collected to isolate lignin samples. For example, black liquor from raw and HTP tobacco stalks was dropped into the acidic water (pH 2) under continuous magnetic stirring to precipitate BLs, which were labeled as BL_R_ and BL_HT_, respectively. Parallelly, the native lignin milled wood lignin (MWL) from raw tobacco stalk was also isolated to investigate the structural changes of lignin during DES deconstruction and alkaline delignification (Björkman, 1954).

### Analysis methods of the lignin samples

In order to comprehensively compare the macromolecular structural characteristics of lignins, different characterization techniques were applied. Gel permeation chromatography (GPC, Agilent 1200, United States) equipped with a refractive index detector was used to detect the number-average (Mn) and weight-average (Mw) molecular weights of the lignin fractions (Wang et al., 2017). The Bruker AVIII 400 MHz spectrometer (Bruker, Germany) was adopted to collect the quantitative 2D-HSQC spectra of the lignin dissolved in DMSO-*d*_6_ (Rencoret et al., 2008; Wen et al., 2013a; Wang et al., 2017). Thermal stability and functional characteristics (i.e., UV-vis absorption) of these lignin samples were also assessed with a DTG analyzer (DTG-60, Shimadzu, Japan) and UV spectroscopy (UV-2550, Shimadzu, Japan) as previously (Wang et al., 2022). In addition, a scanning electron microscope (SEM, SU8010) was selected to observe the microscopic morphology of the recovered lignin fractions to guide their further application.

## Results and discussion

### Yields and molecular weight distributions of lignin

Lignin yield (based on Klason lignin) is an important criterion for assessing the effectiveness of the lignocellulose deconstruction, which is also crucial for the structural characterization of lignin. MWL is the typical native lignin that can reveal the representative structures of raw lignin samples from lignocellulose. In this study, all the yields of the lignin samples are listed in Table 1. MWL presented the lowest yield of 35.6%, which prominently originated from the high recalcitrance of raw tobacco stalk (Pu et al., 2013). In contrast, lignin samples from DES deconstruction and alkaline delignification showed a significant enhancement (ranging 50.4%–80.3%) compared to that of MWL, indicating that these deconstruction processes were efficient for tobacco stalk, and the biomass recalcitrance was effectively disrupted. Among them, alkaline delignification processes dissociate the higher amount of lignin, suggesting that traditional alkaline pulping is an efficient pathway for lignocellulose delignification. BL_HT_ showed a higher yield than BL_R_ due to the pre-subjected HTP process that can partially dissociate the rigid matrix of biomass (Li et al., 2018). Similarly, an identical phenomenon was also observed in DES lignins (Table 1). Furthermore, the recovered yield of lignin from the ChCl/EG system was slightly higher than that of the ChCl/Gly system, indicating that ChCl/EG exhibited more obvious delignification efficacy for this tobacco stalk. In addition, the sugar contents in the lignin fractions showed that BLs had more carbohydrates than those of DES lignins, which also can be verified by the following 2D HSQC spectra.

**TABLE 1:** Yields and weight-average (Mw), number-average (Mn) molecular weights, and polydispersity index (PDI, Mw/Mn) of lignin samples (g/mol).

Sample	Yield (%)	Sugar content (%)	Mw	Mn	PDI
MWL	35.6 ± 1.1	3.4	2,580	2,010	1.3
L_EG_	56.5 ± 0.8	1.5	3,050	3,000	1.0
L_Gly_	50.3 ± 1.2	3.1	2,410	2,350	1.0
HL_EG_	65.1 ± 0.9	0.8	2,130	1,710	1.2
HL_Gly_	53.4 ± 1.5	2.8	1,880	1,860	1.0
BL_R_	74.6 ± 1.5	3.5	2,470	2,370	1.1
BL_HT_	80.3 ± 1.8	2.0	2,790	2,800	1.0

Molecular weights of different lignin samples have also been recorded by the GPC technique, and the relative results are listed in Table 1, in which L_EG_ and L_Gly_ showed larger molecular weights (3,050 and 2,410 g/mol, respectively) than HL_EG_ and HL_Gly_ (2,130 and 1,880 g/mol, respectively). In fact, the HTP process plays a vital role in depolymerizing lignin polymer that partially reduces the molecular weights of HL_EG_ and HL_Gly_ (Li et al., 2018; Wang et al., 2019a). Moreover, BLs showed relatively minor molecular weights suggesting that lignin macromolecule was also greatly depolymerized during the alkaline cooking procedure. Notably, the PDI of all the recovered lignin samples narrowly ranged 1.2–1.0. This implied that these lignin fractions were extremely homogenous polymers that can be targeted into various lignin-based products, such as lignin-derived functional or nanomaterials (Sajjadi et al., 2021; Wang et al., 2021). It was reported that the lignin with lower molecular weights normally contained significant amounts of functional groups, such as hydroxyl and carboxyl groups (Laurichesse and Averous, 2014), and these characteristics will encourage the upgrading of the lignin polymers.

### 2D-HSQC NMR spectra of lignin

**FIGURE 2:**
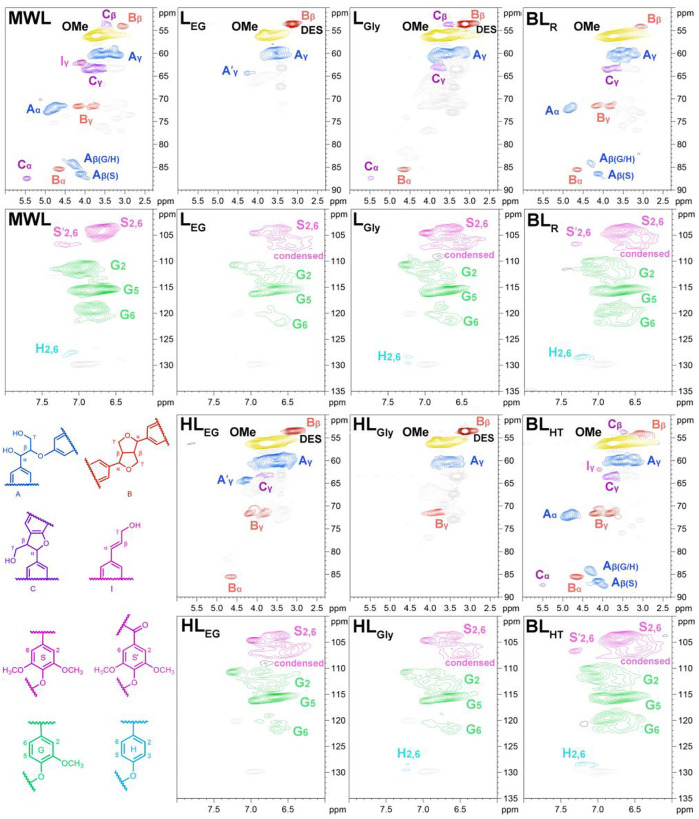
2D-HSQC spectra of different lignin samples and the identified linkages.

The advanced 2D-HSQC NMR technique was applied to characterize lignin (Rencoret et al., 2008; Wen et al., 2013b). The obtained 2D-HSQC spectra of these lignin samples from tobacco stalk are presented in Figure 2, and the quantitative results of linkages are also listed in Table 2.

**TABLE 2:** Quantification of lignin samples by the quantitative 2D HSQC method.

Sample	β-*O*-4	β-β	β-5	S/G
MWL	49.5	8.0	5.2	0.5
L_EG_	ND	ND	ND	0.5
L_Gly_	ND	3.3	1.0	0.6
HL_EG_	ND	ND	ND	0.6
HL_Gly_	ND	ND	ND	0.5
BL_R_	13.4	4.2	0.4	0.6
BL_HT_	17.5	5.1	0.7	0.9

The side-chain region of 2D-HSQC spectra from these lignin samples showed the diverse linkages present in the lignin macromolecules (Figure 2). For the MWL from tobacco stalk, a typical 2D-HSQC spectrum of native lignin was observed that the main linkages, such as β-*O*-4 linkages (A), β-β linkages (B), and β-5 linkages (C), with three obvious chemical shifts (α, β, and γ positions), can be clearly assigned based on previous literature (Wen et al., 2013a; Wang et al., 2017; Wang et al., 2022). Then, *p*-hydroxycinnamyl alcohol end groups (I) also can be identified with strong signal intensity. However, the lignin fractions from DES pretreatment and alkaline cooking showed severely destroyed structures, in which most of the linkages were disrupted and disappeared in the spectra (especially for DES lignins), which definitely resulted from the crushing conditions during these delignification processes (Ma et al., 2022). It was also noted that BLs showed a relatively integrated structure compared to other DES lignins. This phenomenon can be attributed to the fact that aqueous alkaline solvent has an excellent swelling effect on lignocellulose and has little effect on the lignin structures (typical substructures), leading to more lignin fractions efficiently dissolved and presenting high structural intensity (Wen et al., 2015; Sun et al., 2019). Furthermore, the γ-acetylated β-*O*-4 linkages (A′) occurred in the 2D-HSQC spectra of L_EG_ and HL_EG_, owing to the acylation reaction that occurred in the lignin side chain under the given conditions.

In the aromatic region of 2D-HSQC spectra (Figure 2), signals from the syringyl (S), guaiacyl (G), and *p*-hydroxyphenyl (H) units were clearly distinguished in MWL (Rencoret et al., 2008). After the delignification process, the recovered lignin showed a relatively lower signal intensity and obvious condensed signals in S units (such as L_EG_ and HL_EG_). It was found that H units disappeared in L_EG_ and HL_EG_, and different reductions of signal intensities were also observed in other lignin samples. As mentioned earlier, BL_R_ and BL_HT_ also presented the relative integrity signals that the main S, G, and H units still contained, except for the occurrence of condensed signals at S units. In fact, condensation reaction also occurred in G units, as proved in the 2D-HSQC spectra (Figure 2). It has been reported that condensation reactions occur not only at the 2, 5, and 6 positions of G units but also happen at the 5-position of other aromatic rings and Cα of lignin side-chains (Torr et al., 2012; Shen et al., 2019). In this study, the chemical shifts of G units (especially G2 and G6) were changed during DES pretreatment, which resulted from the formation of intricate C-C linkages during acid-catalyzed condensation (Torr et al., 2012). Additionally, it was noted that these lignin samples isolated from tobacco stalk showed no *p*-hydroxycinnamate (PCA and FA) structures, which is similar to hardwood lignin (SGH-type lignin) (Rencoret et al., 2008; Sun et al., 2019).

**SCHEME 1:**
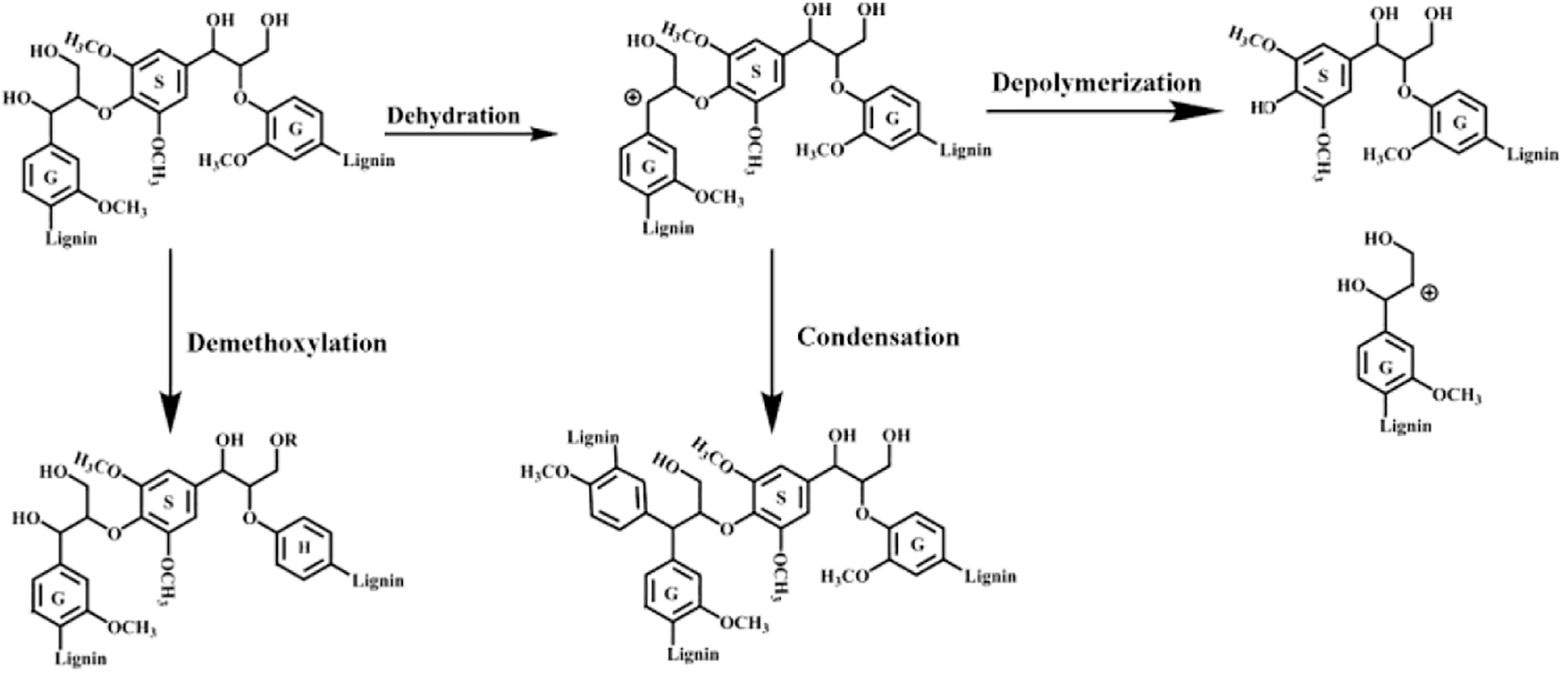
Potential pathway for the lignin structural transformation during the delignification.

The quantitation of these 2D-HSQC spectra was acquired and presented in Table 2. As expected, the content of β-*O*-4 linkage in MWL was abundant compared to the other recovered lignin, which was 49.5/100 Ar. Also, BL_R_ and BL_HT_ exhibited minor amounts of β-*O*-4 linkages (13.4 and 17.5/100 Ar) that confirmed their structural integrity, as reflected in Figure 2. Lignin macromolecule with more β-*O*-4 linkage usually has a larger molecular weight and relatively intact structure. In the present study, MWL exhibited ideal structural integrity, and the molecular weight was similar to BL_R_ (2,580 vs*.* 2,470 g/mol), while BL_HT_ exhibited a higher molecular weight (2,800 g/mol) that mostly was attributed to the severe condensation reaction of lignin during the HTP process. For the DES lignin fractions, no visible linkages can be detected and only trace amounts of C-C linkages (e.g., β-β and β-5) were found in L_Gly_ (Table 2). In this study, MWL and DES lignin samples exhibited similar S/G ratio values even if the treated lignins had no raw linkages, and this result was caused by the obvious condensation that the units were linked by C-C linkages during the harsh condition. However, the higher S/G ratio values of BLs could be explained by demethoxylation during the alkali delignification (Wang et al., 2019b). Scheme 1 lists the potential pathway for the lignin structural transformation during this delignification (Shen et al., 2019; Wang et. al., 2019a).

### Micromorphology of the lignin

**FIGURE 3:**
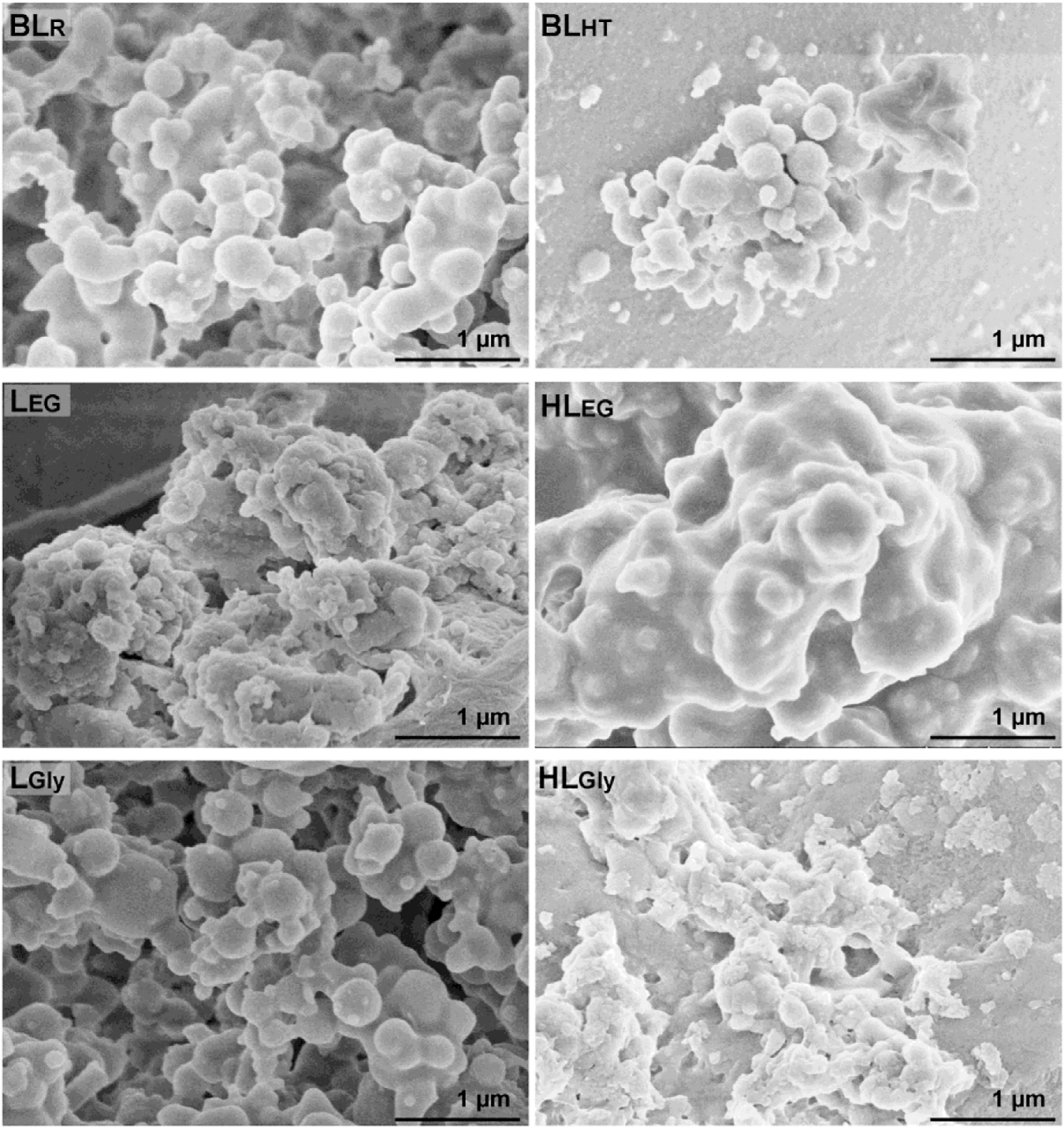
SEM images of sub-micron spheres from recovered lignin fractions.

Recently, lignin nanoparticles or colloidal lignin particles have attracted increased attention, and some reports envision that lignin nanoparticles will have an important influence on promoting lignin valorization, such as the development of synthetic nanoparticles in the polymer industry (Zhao et al., 2016). Indeed, lignin micro-nanoparticles also possess outstanding antioxidant, UV-absorbing, and antimicrobial properties, which can be converted into sustainable biomedical materials, nanocomposites, and functional materials (Wang H et al., 2018; Monika et al., 2020; Siavash and Rajender, 2020). In this study, the micromorphology of the lignin fractions was investigated by scanning electron microscope (SEM), and the SEM images of sub-micron lignin spheres are presented in Figure 3. It can be observed that BLs presented some regular micro-nanoparticles with different sizes, suggesting that lignin was well self-assembled during the recovery process from black liquor (Xu et al., 2022). Then, the recovered DES lignin also exhibited a micro-nanoparticle shape, in which the L_EG_ and L_Gly_ showed a relatively regular and large micro-nanoparticle shape. However, the HL_EG_ and HL_Gly_ presented the remarkable agglomeration of lignin. The severe agglomeration phenomenon of HL_EG_ and HL_Gly_ is probably owing to the HTP process that resulted in the condensed reaction of lignin (especially for HL_EG_). Furthermore, HL_Gly_ also showed the smaller size of nanoparticles, which was in line with its lower molecular weight, as stated in Table 1. All the aforementioned results confirmed that the inherent structures of lignin would play a crucial role in the formation of lignin microtopography during the recovery procedure. Therefore, these DES lignins and BLs are exactly promising in fabricating lignin nanoparticles and lignin-based materials, which will deeply promote lignin conversion in the current green and sustainable biorefinery.

### FTIR analysis of lignin

**FIGURE 4:**
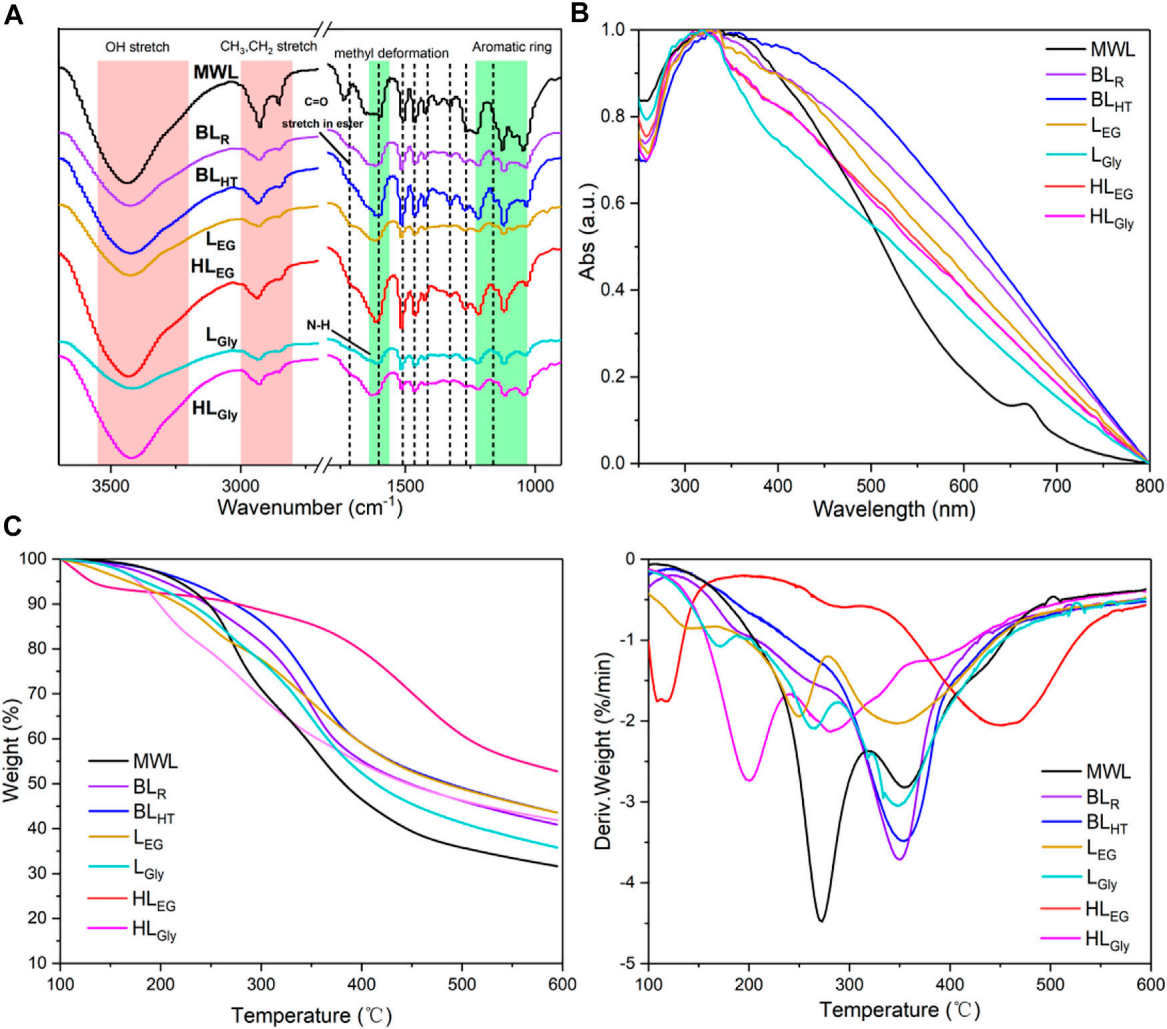
**(A)** FTIR spectra, **(B)** UV–vis absorption spectra, and **(C)** TG/DTG curves of the different lignin samples.

Figure 4A illustrates the FTIR spectra of all the lignin samples, which were assigned based on the previous literature (Dong et al., 2019). It could be observed that the peaks ranging 1,350–1,050 cm^−1^ of the FTIR spectra were attributed to the signals’ vibration of the aromatic ring in lignin. The absorption peak located at 1,162 cm^−1^ is responsible for the esterified lignin (Christopher et al., 2019; Zhong et al., 2021), and MWL showed a strong absorption peak here, suggesting that more esterified linkages exist in MWL; that is, MWL had a high structural intensity. Next, the absorption peaks at 1,603, 1,510, 1,465, and 1,415 cm^−1^ were also attributed to the aromatic ring vibration and methyl deformation of lignin. Among them, the spectra of MWL exhibit a strong absorption peak around 1,750 cm^−1^ that originated from the C=O stretch ester of native lignin. Obviously, the abundant peaks at 2,800–3,000 cm^−1^ for methyl and methylene groups of the MWL skeleton could be easily identified as compared with the other lignins, and this result indicated that the lignin polymer was significantly depolymerized during these DES pretreatment and alkaline delignification processes (Wang et al., 2022). In this study, lignin was extracted from tobacco stalk by the effective cleavage of lignin–carbohydrate linkages (LCC) and then dissolved in DESs and alkaline solvents (Wang et al., 2019b; Shen et al., 2019). In practice, some chemical reactions (e.g., depolymerization and condensation) occurred in lignin macromolecule and resulted in the more complex structures under given conditions (Zhong et al., 2021), which will restrict their utilization and targeted conversion. Therefore, exploring the applicable properties of the lignin is of significance for its value-added valorization, such as abundant functional groups, UV absorption, and high thermostability (Wang et al., 2022).

### Ultraviolet light absorption and thermal stability of lignin

The existence of functional groups in lignin, such as phenolic hydroxyl groups and aromatic rings or conjugated double bonds, fatefully endows lignin with brilliant UV light absorption that could largely enhance the scope of practical application. Naturally, the UV absorption features of the lignin are also related to its macromolecular structure (Wang et al., 2022). In this study, the UV–vis absorption spectra of all the lignin samples were acquired, as shown in Figure 4B; it can be observed that all the lignin samples showed prominent absorption capacity toward UV light, suggesting that these have ideal UV absorption property. MWL showed the weaker absorption property was mostly related to its less functional groups (e.g., hydroxyl groups) in lignin polymer than that of other lignin fractions. Interestingly, BL_R_ and BL_HT_ exhibited the analogical spectra toward light absorption, and a similar phenomenon was also noticed for HL_EG_ and HL_Gly_. The aforementioned results subtly indicated that lignin possessed identical structures that would show similar UV–vis absorption (Wang et al., 2022), whereas L_EG_ and L_Gly_ had different spectra mostly owing to their structural differences in the abundances of linkages and units, as shown in Figure 2. The promising UV absorption features of these lignin polymers revealed significant prospects in producing lignin-based UV-shielding materials (Monika et al., 2020; Wang et al., 2020), which could boost the upgrading feasibility of lignin in a sustainable pathway.

It has been reported that lignin structural characteristics play an important role in subsequent conversion and material properties of products, such as functional or carbon materials (Zhang et al., 2019; Xi et al., 2020b; Wang et al., 2021). Hence, investigating the thermal stability of lignin is vital for its subsequent conversion into value-added carbon materials and thermoplastic materials (Sanghamitra et al., 2015). The present study analyzed all the lignin samples by thermogravimetric analysis (TGA) to reveal the correlation between structural characteristics and thermal stability. Figure 4C shows that TG/DTG curves of the lignins are presented. Notably, the thermal degradation behavior of different lignin samples was diversiform, which was influenced by their inherent structures, that is, the chemical linkages, the abundance of functional groups, and the degree of condensation (Liu et al., 2021; Wang et al., 2022). It has been reported that the thermal decomposition behavior of lignin polymer was correlated to its molecular weight, whereas an inconspicuous tendency was found in this study, and this could be explained by their little differences in molecular weights (Table 1). Detailedly, MWL had the lowest content of residual char compared to the other treated lignin fractions, as shown in TGA (Figure 4C, left), which can be attributed to its abundant ether bonds and minor condensed linkages. It was observed that a slight reduction of weight before 200°C occurred in these lignin samples owing to the dehydration of the lignin macromolecule and weak or labile linkages disrupted. Then, the mass loss of lignins was drastic with the temperature increased up to 350°C. This change may be attributed to the side chain oxidation of lignin under high temperatures. As the temperature further enhanced, the rates of mass loss of lignin samples were visibly weak compared to the front period, in which C–C bonds and aromatic ring collapsed and sedimented into residual char. Finally, it was noticeable that almost lignin fractions showed a relatively high content of residual char (exceeding 40%), implying that these lignin polymers are excellent feedstock for the fabrication of carbon materials.

## Conclusion

The present study adopted DES and alkaline delignification to deconstruct tobacco stalk before and after hydrothermal pretreatment, achieving different lignin fractions with various structural characteristics. Two kinds of DES isolated lignin polymer with higher yields and homogenous molecular structure compared to MWL. DES deconstruction of tobacco stalks resulted in the obvious disruption of linkages in lignin compared to those of black liquor lignins (BLs), mostly owing to the efficient delignification of DES and ideal dissolution of lignin in the alkaline solvent. Moreover, the recovered lignin fractions exhibited good micro-nanoparticle size that will significantly enhance the valorization of lignin in fabricating nanomaterials, in which the lignins from hydrothermal-assisted DES deconstruction presented the smaller nanoparticle size that mostly originated from their reduced molecular weights. Furthermore, all the recovered lignin showed excellent UV absorption and had a structure-induced absorption performance or carbon content. All these features promote these lignin fractions serving as great feedstock for producing sustainable functional or carbonaceous materials.

## Data Availability

The original contributions presented in the study are included in the article/supplementary material. Further inquiries can be directed to the corresponding authors.
